# Therapeutic Effect of Arginine, Glutamine and β-Hydroxy β-Methyl Butyrate Mixture as Nutritional Support on DSS-Induced Ulcerative Colitis in Rats

**DOI:** 10.3390/nu18020208

**Published:** 2026-01-09

**Authors:** Elvan Yılmaz Akyüz, Cebrail Akyüz, Ezgi Nurdan Yenilmez Tunoglu, Meryem Dogan, Banu Bayram, Yusuf Tutar

**Affiliations:** 1 Department of Nutrition and Dietetics, University of Health Sciences, 34668 Istanbul, Türkiye; elvan.yilmazakyuz@sbu.edu.tr (E.Y.A.); banu.bayram@sbu.edu.tr (B.B.); 2 Department of General Surgery, Haydarpasa Numune Training and Research Hospital, University of Health Sciences, 34668 Istanbul, Türkiye; drcakyuz@hotmail.com; 3 Division of Medical Techniques and Services, Vocational School of Health Services, Demiroglu Science University, 34394 Istanbul, Türkiye; ezgi.tunoglu@demiroglu.bilim.edu.tr; 4 Department of Pathology, Haydarpasa Numune Training and Research Hospital, University of Health Sciences, 34668 Istanbul, Türkiye; m.beradogan@hotmail.com; 5 Division of Biochemistry, Faculty of Pharmacy, University of Health Sciences, 34668 Istanbul, Türkiye; 6 Division of Medicinal Biochemistry, Faculty of Medicine, Recep Tayyip Erdogan University, 53020 Rize, Türkiye; 7 Training and Research Hospital, Recep Tayyip Erdogan University, 53020 Rize, Türkiye; 8 Medical Oncology Program, Recep Tayyip Erdogan University, 53100 Rize, Türkiye; 9 Molecular Medicine Program, Recep Tayyip Erdogan University, 53100 Rize, Türkiye

**Keywords:** inflammatory bowel disease, DSS colitis, arginine, glutamine, HMB, nutrigenomics, cytokine signaling

## Abstract

Background: Ulcerative colitis (UC) is characterized by chronic mucosal inflammation, oxidative stress, and disruption of intestinal metabolic homeostasis. Immunomodulatory nutrients such as arginine, glutamine, and β-hydroxy β-methylbutyrate (HMB) have shown potential benefits; however, their combined molecular effects on UC remain insufficiently defined. Objective: To investigate the individual and combined effects of arginine, glutamine, and HMB on inflammatory and metabolic gene expression, oxidative stress markers, and histopathological outcomes in a dextran sulfate sodium (DSS)-induced colitis model. Methods: Female Sprague Dawley rats were assigned to six groups: control, DSS, DSS + arginine, DSS + glutamine, DSS + HMB, and DSS + mixture. Colitis was induced using 3% DSS. Colon tissues were examined histologically, serum MDA, MPO, and GSH levels were quantified, and mRNA expression of *IL6*, *IL10*, *COX2*, *NOS2*, *ARG2*, *CCR1*, and *ALDH4A1* was measured by RT-qPCR. Pathway enrichment analyses were performed to interpret cytokine and metabolic network regulation. Results: DSS induced severe mucosal injury, elevated MDA and MPO, reduced GSH, and significantly increased *IL6*, *COX2*, *NOS2*, *ARG2*, and *CCR1* expression. Glutamine demonstrated the strongest anti-inflammatory and antioxidant effects by decreasing *IL6* and *COX2* and restoring GSH. Arginine primarily modulated nitric oxide–related pathways, whereas HMB increased *ALDH4A1* expression and metabolic adaptation. The combination treatment produced more balanced modulation across inflammatory, chemokine, and metabolic pathways, consistent with enrichment results highlighting cytokine signaling and amino acid metabolism. Histopathological improvement was greatest in the mixture group. Conclusions: Arginine, glutamine, and HMB ameliorate DSS-induced colitis through coordinated regulation of cytokine networks, oxidative stress responses, and metabolic pathways. Their combined use yields broader and more harmonized therapeutic effects than individual administration, supporting their potential as targeted immunonutritional strategies for UC. Rather than targeting a single inflammatory mediator, this study was designed to test whether combined immunonutrient supplementation could promote coordinated regulation of cytokine signaling, oxidative stress responses, and metabolic adaptation, thereby facilitating mucosal repair in experimental colitis.

## 1. Introduction

Inflammatory bowel disease (IBD) is a functional gastrointestinal disorder of unknown etiology characterized by acute and chronic inflammation of the gastrointestinal tract, with periods of recurrent relapse and remission [1]. IBD is increasing worldwide. The global age-standardized incidence rate of IBD is 4.45 per 100,000 and is more common in women. The burden of IBD is expected to increase as the world’s population grows and ages [2]. Possible mechanisms and mediators that cause disease progression are still being investigated, and the underlying mechanism is thought to be dysregulation of inflammatory processes involving proinflammatory cytokines [3].

Inflammatory bowel diseases are a group of diseases in which malabsorption of nutrients and malnutrition occur in 20–85% of patients [4,5]. The disease is primarily treated with drugs, but the pharmacological agents used increase the risk of anemia and malignancy due to the development of opportunistic infections [6]. Malnutrition leads to bacterial translocation and disruption of the gastrointestinal tract mucosal barrier integrity. To prevent protein loss due to ulceration and to alleviate symptoms, it is necessary to replace nutritional deficiencies [3,7]. Dietary modulation can reduce disease-related symptoms in IBD, prevent relapses, and contribute to successful management of the disease [7,8]. Currently, there is no accepted nutritional treatment for patients with IBD.

Proteins are important in the management of IBD, its remission, the reduction in its complications and the improvement of the quality of life of individuals [7]. Pharmacological agents can alter amino acid metabolism. It is noteworthy that the amino acid requirements of patients with IBD differ from those of healthy individuals [9]. Glutamine and arginine have significant effects on the immune system and may be a potential treatment for IBD [10]. Arginine is a conditionally essential amino acid and a precursor of NO. It causes a decrease in the number of suppressor T cells, an increase in the number of helper T cells, and an increase in the lymphatic response to mitogens. Tissue arginine levels are reduced in IBD due to decreased uptake and increased *NOS2* [11]. Glutamine is a precursor for the biosynthesis of nucleic acids and many biologically important molecules [9]. Therefore, it is an essential amino acid for the continuity of gastrointestinal mucosa, lymphocytes and fibroblasts, which are constantly dividing and multiplying. It is necessary for maintaining the integrity of the intestinal structure, but its role in IBD is not clear [12]. HMB is an active metabolite of the essential amino acid leucine. It is synthesized in small amounts in humans and has an effect on the immune system. HMB has anti-inflammatory effects in the gut and may be a potential therapeutic candidate for the treatment of IBD [13].

Arginine, glutamine, and β-hydroxy β-methylbutyrate (HMB) were selected based on their complementary roles in intestinal immunity and metabolism. Glutamine supports epithelial integrity and antioxidant capacity; arginine regulates nitric oxide–dependent immune responses; and HMB, a leucine-derived metabolite, has been reported to influence inflammatory signaling and cellular metabolic responses and to support tissue repair processes, particularly under conditions of inflammatory or catabolic stress [14,15,16,17]. Together, these nutrients represent a targeted immunonutritional approach aimed at restoring mucosal homeostasis through coordinated regulation of inflammatory and metabolic pathways.

The demonstration of the effect of the arginine + glutamine + HMB mixture used in enteral nutrition therapy on gastrointestinal tissue damage caused by IBD suggests that the mixture may have an alternative use in the treatment of IBD. To our knowledge, no study has been conducted to investigate the effects of this mixture on the molecular level against damage caused by experimental intestinal colitis. The aim of this study is to investigate the mechanisms involved in the arginine + glutamine + HMB mixture in DSS-induced colitis. Elucidation of these mechanisms will provide a basis for development of new treatment strategies.

## 2. Materials and Methods

Animals and Chemicals: The study protocol was approved by the University of Health Sciences Local Animal Experiments Ethics Committee. Following approval of the experimental protocol, 48 female Sprague Dawley rats, 8–12 weeks old (200–300 g), were obtained from SBU Laboratory Animal Center in Istanbul, Türkiye (Decision no: 2018-02/03, 3 April 2018). Only female Sprague Dawley rats were used in this study to reduce variability related to sex-dependent differences in DSS-induced colitis severity and inflammatory responses. This approach is consistent with previous DSS colitis models and reflects the higher prevalence of ulcerative colitis reported in female populations. Rats were divided into six groups: control (*n* = 8), colitis (*n* = 8), colitis with l-arginine supplementation (DSS + Arginine; *n* = 8), colitis with l-glutamine supplementation (DSS + Glutamine; *n* = 8), colitis with β-hydroxy-β-methylbutyrate butyrate supplementation (DSS + HMB; *n* = 8) and colitis with arginine, l-glutamine and β-hydroxy-β-methylbutyrate mix supplementation (DSS + Combine; *n* = 8). A total of 48 rats were housed in cages with four rats per cage. The rats were kept in a standard laboratory environment with a 12 h light/dark cycle, 45–60% relative humidity, and room temperature of 22 ± 2 °C.

DSS was acquired from Sigma-Aldrich Chemical Co. (St. Louis, MO, USA) and was used in this study to induce colitis in rats. L-glutamine was obtained from Nestle Health Science (Istanbul, Türkiye). L-Arginine was bought from Sepe Natural Co. (Izmir, Türkiye). HMB was purchased from Zhangjiagang Specom Biochemical (Zhangjiagang, China). Arg/Glu/HMB mixture was bought from Abbott Nutrition (Istanbul, Türkiye).

Experimental Design: Animals were divided into six groups. For seven days, animals were fed a standard diet and drinking water ad libitum. Group 1 (control) received the standard diet without DSS. Group 2 (colitis) received the standard diet with DSS. Colitis was induced in the groups 2, 3, 4, 5, and 6 by administering DSS (3% [*w*/*v*], molecular weight 40,000) in the drinking water for the 7 days. Following DSS induction, supplements were administered orally twice daily (morning and evening) from day 8 to day 14 using a sterile 4F flexible polyurethane feeding tube (outer diameter ≈ 1.3 mm), gently inserted via the oral cavity into the esophagus. Rats were briefly restrained manually without anesthesia, and correct placement was ensured prior to administration to prevent tracheal insertion. Each dose was freshly prepared in distilled water and delivered in a total volume not exceeding 1 mL per administration. After colitis was induced, the group 3 received 200 mg/kg l-arginine, the group 4 received 200 mg/kg l-glutamine, the group 5 received 40 mg/kg β-hydroxy-β-methyl methyl butyrate, and the group 6 received an Arg/Glu/HMB mixture containing 200 mg/kg arginine, 200 mg/kg glutamine, and 40 mg/kg β-hydroxy-β-methyl methyl butyrate orally twice a day via a 4F feeding tube between the 8th and 14th days. The dosage of supplements was determined based on the previous study [18]. On day 15, rats were sacrificed under general anesthesia induced by intramuscular injection of ketamine (100 mg/kg) and xylazine (10 mg/kg), and then the colon and blood samples were taken. The colonic segment was used for histopathological observation. The proximal segment was used for PCR analysis. Additionally, body weights were recorded daily during the experiment.

Morphology and Histological Evaluation: Colon tissues were removed and fixed in 10% neutral formalin. Then they were embedded in paraffin and stained with hematoxylin and eosin (HE). The Nancy index was used to evaluate histological scoring of colitis. It evaluates three key features of mucosal activity: chronic inflammatory infiltration, acute inflammatory infiltration, and ulceration. Each component is scored on a five-grade scale ranging from 0 (no or minimal histological activity) to 4 (severe disease) [19]. The stained sections were photographed and observed using an Olympus CX41 light microscope (Tokyo, Japan).

Tissue Collection and RNA Isolation: In accordance with the experimental protocol, animals were euthanized and colon tissues were removed. The tissues were washed gently using ice-cold phosphate-buffered saline (PBS) to clear any luminal contents. A gentle wash with ice-cold phosphate-buffered saline (PBS) was carried out, followed by snap-freezing in liquid nitrogen to maintain RNA integrity. Total RNA was extracted from approximately 30 mg of frozen tissue using TRIzol™ reagent (Invitrogen, Carlsbad, CA, USA) following the manufacturer’s protocol. The quantity and quality of RNA were assessed using NanoDrop spectrophotometer (Thermo Fisher Scientific, Waltham, MA, USA), and only samples with A260/A280 ratios of 1.8 to 2.0 were acceptable for downstream applications.

cDNA Synthesis and Quantitative Real-Time PCR (RT-qPCR): The High-Capacity cDNA Reverse Transcription Kit (Euroclone, Pero, Italy) allowed for the reverse transcription of high-quality RNA into cDNA. Quantitative real-time PCR was performed with SYBR Green PCR Master Mix (SensiFAST, Bioline, Meridian Bioscience, Cincinnati, OH, USA). The PCR conditions included an initial 5 min denaturation step at 95 °C, followed by 40 cycles of 15 s at 95 °C and 30 s at 60 °C. The expression levels of the genes were determined and analyzed with the 2^−ΔΔCt^ method. To study the effect of experimental colitis on immune-related molecular pathways, a custom designed 96-well qPCR array was performed with the aim of screening altered expressions of selected genes. The array provides a comprehensive overview of inflammatory processes by profiling genes associated with chemokines, cytokines, and their receptors, pertinent signaling pathways, apoptosis, immune regulation, and glutamic acid metabolism. Housekeeping gene *ACTINB* was included for normalization purposes. To achieve technical reproducibility, all samples were run in triplicates [20].

Serum Collection and ELISA Assays: Blood collection through cardiac puncture was followed by centrifugation at 3000× *g* for 10 min at 4 °C to separate serum samples. The analysis of serum levels of malondialdehyde (MDA), myeloperoxidase (MPO) and reduced glutathione (GSH) was performed through ELISA assays utilizing Elabscience R&D commercial kits (Elabscience Biotechnology, Wuhan, China) that followed manufacturer instructions. A microplate reader (Thermo Fisher Scientific operated in the USA) measured absorbance values at specified wavelengths. Each sample underwent duplicate processing for analysis.

Gene and Metabolite Enrichment Analysis: EnrichR (https://maayanlab.cloud/Enrichr/, accessed on 10 November 2025) and Metaboanalyst (https://www.metaboanalyst.ca/) enrichment tools were employed. These tools provide a systems-level understanding of colitis by identifying coordinated disruptions in inflammatory signaling, amino acid metabolism, mitochondrial function, and oxidative stress pathways. These analyses reveal mechanistic relationships that explain how immunonutrients such as arginine, glutamine, and HMB modulate *IL-10* signaling, CD163-mediated anti-inflammatory responses, and cytokine networks. Enrichment tools therefore bridge molecular data with biological interpretation, supporting the development of targeted nutritional strategies for colitis.

The selected gene and biochemical markers were chosen to reflect key inflammatory, chemokine, oxidative stress, and metabolic pathways known to be dysregulated in DSS-induced colitis and directly influenced by arginine, glutamine, and HMB metabolism. This targeted approach allowed evaluation of coordinated immunometabolic responses rather than isolated inflammatory endpoints.

Statistical Analysis: The data presentation used mean values together with standard deviation (SD) as measurement. Only mRNA expression alterations showing more than a twofold upregulation or downregulation (fold change thresholds >2 or <−2) were interpreted as biologically significant. Statistical analyses were performed with SPSS v26.0 (IBM Corp., Armonk, NY, USA), using Student’s *t*-test for comparisons; *p* < 0.05 was considered statistically significant. Statistical significance was determined by one-way ANOVA followed by post hoc Tukey’s HSD analysis; *p* < 0.05 vs. DSS-colitis group.

## 3. Results

### 3.1. Body Weight

**Figure 1:**
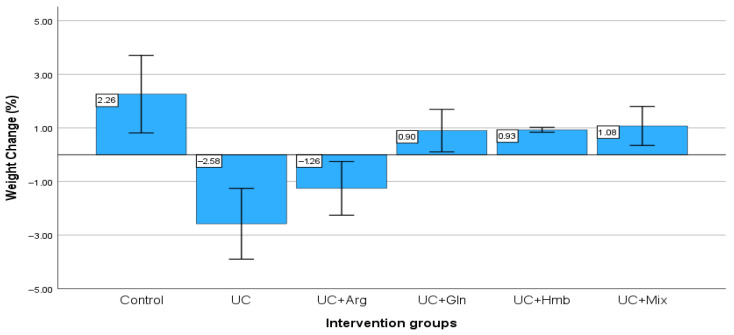
% Body Weight Change (%Δ). The most significant weight loss was observed in the UC group, while the arginine group also lost weight but had a significantly higher weight than the UC group. The glutamine, HMB, and mixture groups showed an increase in body weight similar to that of the control group. Paired *t* test was used and then analyzed using ANCOVA (*p* < 0.001).

When the initial and final body weights of the groups were examined in the study, significant weight loss was observed in the UC and UC + arg groups (*p* < 0.001), while a statistically significant increase was found in all other groups (*p* < 0.001). The UC group showed the lowest final weight. The final weight values of the UC + Gln, UC + Hmb, and UC + Mix groups were found to be similar to those of the control group (Figure 1).

### 3.2. Histopathological Findings

**Figure 2:**
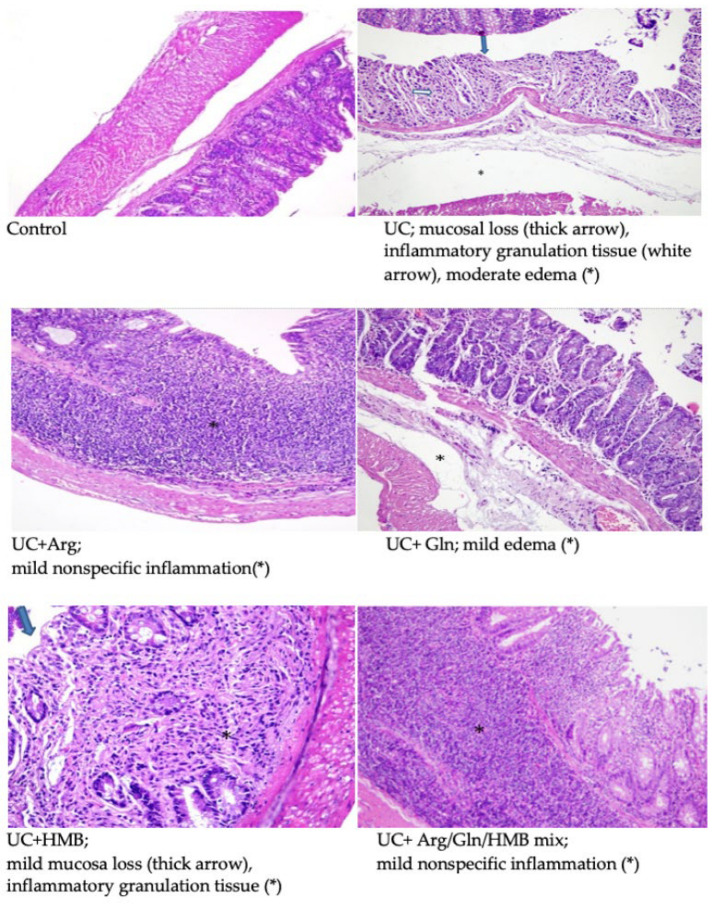
Colon microscopic structure of rats, H&E, 200×. Compared to the control group, the DSS-induced colitis group exhibited epithelial surface ulcerations, increased inflammatory cells in the lamina propria and submucosal edema. While these changes in colitis were reduced in all treatment groups, the greatest improvement in the epithelial barrier occurred in the HMB and mixed groups.

The histological structure and appearance of the rat colons are shown in Figure 2. The colon morphology of the control group is normal. In the colitis group, there is mucosal loss on the surface of the colon mucosa (thick arrow), the presence of inflammatory granulation tissue in the lamina propria (white arrow), and moderate submucosal edema (*). In the UC + Arginine group, mild chronic nonspecific inflammation (*) continued from the lamina propria to the submucosa in the colon mucosa. In the UC + Glutamine group, there is mild submucosal edema (*) in the full-thickness colon mucosa. In the UC + HMB group, there is mild mucosa loss on the surface of the full-thickness colon mucosa (thick arrow) and the presence of inflammatory granulation tissue in the lamina propria (*). There is mild chronic nonspecific inflammation (*) in the full-thickness colon mucosa of the UC + Mix group, which continues from the lamina propria to the submucosa.

### 3.3. Gene Expression Analysis

**Figure 3:**
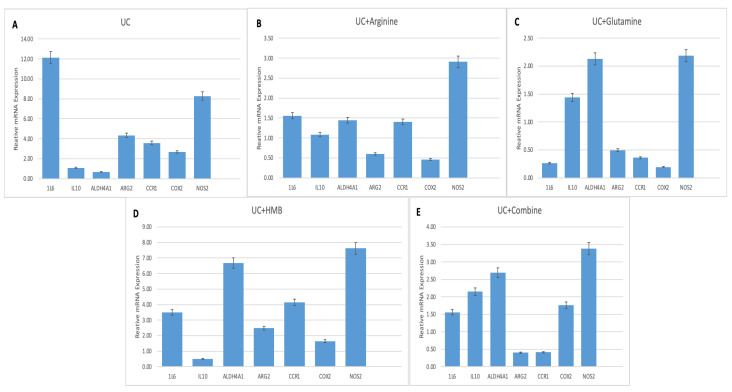
Relative mRNA expression levels of selected genes in colon tissue. Ulcerative colitis (UC) (**A**). UC + Arginine: UC with arginine supplementation (**B**); UC + Glutamine: UC with glutamine supplementation (**C**); UC + HMB: UC with β-hydroxy β-methylbutyrate supplementation (**D**); UC + Combine: UC with combined arginine, glutamine, and HMB supplementation (**E**).

Gene expression analysis revealed key regulatory patterns among treatment groups compared to the DSS-induced colitis group (Figure 3, Table 1). Pro-inflammatory cytokine *IL-6* expression was significantly lower in Glutamine treatment group (0.26-fold, *p* < 0.01), but higher in the UC + Combine (1.56-fold, *p* > 0.05), UC + Arginine (1.56-fold, *p* > 0.05), and UC + HMB (3.51-fold, *p* < 0.01).

The anti-inflammatory cytokine IL10 levels were not significantly changed in UC group (1.09-fold, *p* > 0.05), UC + Arginine (1.09-fold, *p* > 0.05), and UC + HMB (1.0-fold, *p* > 0.05). Conversely, *IL-10* expression levels increased in UC + Glutamine (1.44-fold, *p* > 0.05) and UC + Combine (2.15-fold, *p* < 0.01) significantly.

Involved in various metabolic pathways, *ALDH4A1* levels were significantly higher in UC + HMB (6.68-fold, *p* < 0.01), UC + Combine (2.69-fold, *p* < 0.01), and UC + Glutamine (2.13-fold, *p* > 0.05) compared to the control group. UC + Arginine (1.44-fold, *p* > 0.05) showed no significant difference. Regarding another gene related to metabolic pathways, *ARG2* expression was highest in UC + HMB (2.48-fold, *p* < 0.01), while UC + Combine (0.40-fold, *p* < 0.01), UC + Arginine (0.60-fold, *p* > 0.05), and UC + Glutamine (0.50-fold, *p* < 0.05) all exhibited significant reductions compared to UC.

Regarding immune cell attraction and inflammatory signals, *CCR1* levels were higher in UC + HMB (4.14-fold, *p* < 0.01) and UC + Arginine (1.40-fold, *p* > 0.05), but significantly lower in UC + Glutamine (0.36-fold, *p* < 0.01) and UC + Combine (0.41-fold, *p* < 0.01) compared to the control group.

*COX2* expression did not show a significant increase in any treatment group (*p* > 0.05), and levels were below those in UC across all other treatments. *NOS2* was highest in UC + HMB (7.62-fold, *p* < 0.01), moderately elevated in UC + Arginine (2.91-fold, *p* < 0.05), and UC + Glutamine (2.19-fold, *p* < 0.05), and UC + Combine (3.38-fold, *p* < 0.01).

### 3.4. Serum Oxidative Stress Markers

**Figure 4:**
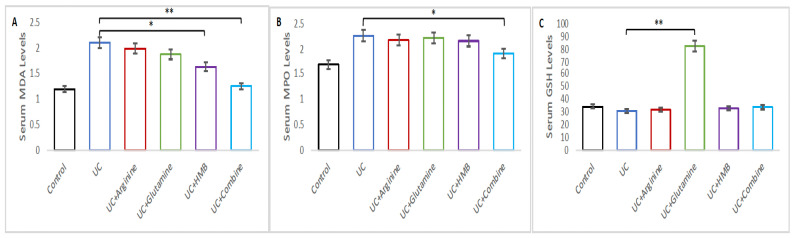
Serum oxidative stress markers measured by ELISA. (**A**–**C**) (**A**) MDA, and (**B**) MPO and (**C**) GSH values of the intestinal tissue in control, UC, UC + Arg, UC + Gln, UC + HMB and UC + Mix groups in DSS induced intestinal mucositis model in rats. Data are presented as mean ± SEM * *p* < 0.05, ** *p* < 0.01.

MDA and MPO levels were elevated in the UC group (*p* < 0.05); however, the GSH level was not significantly changed in the UC group compared with the Control group (*p* > 0.05) (Figure 4).

Serum oxidative stress markers, including malondialdehyde (MDA), glutathione (GSH), and myeloperoxidase (MPO), are shown in Figure 4A–C. For MDA, one-way ANOVA revealed significant differences among groups. Post hoc Tukey’s HSD analysis indicated that the UC + Arginine group (1.98 ± 0.06), UC + HMB group (1.62 ± 0.03), and UC + Glutamine group (1.86 ± 0.07) had significantly lower MDA levels than the UC group (2.09 ± 0.04) (*p* < 0.05). Among the treated groups, we determined the most significant reduction in the UC + Combine group (1.24 ± 0.07), (*p* < 0.01).

MPO results showed that the UC + Arginine group (2.21 ± 0.04), UC + Glutamine group (2.04 ± 0.09), and UC + HMB group (2.17 ± 0.06) had lower levels than the UC group (2.25 ± 0.02), but not statistically significant (*p* > 0.05). However, the UC + Combine group (2.04 ± 0.08) was significantly reduced compared to the UC group (2.25 ± 0.02) (*p* < 0.05).

GSH levels increased in the UC + Arginine (32.03 ± 0.08), UC + HMB (32.92 ± 0.05), and UC + Combine groups (33.88 ± 0.10), with no statistically significant differences (*p* > 0.05). Notably, the UC + Glutamine group displayed a markedly elevated GSH concentration (81.89 ± 0.02), representing a highly significant increase compared with all other groups (*p* < 0.01).

### 3.5. Gene and Metabolite Enrichment Analysis

**Figure 5:**
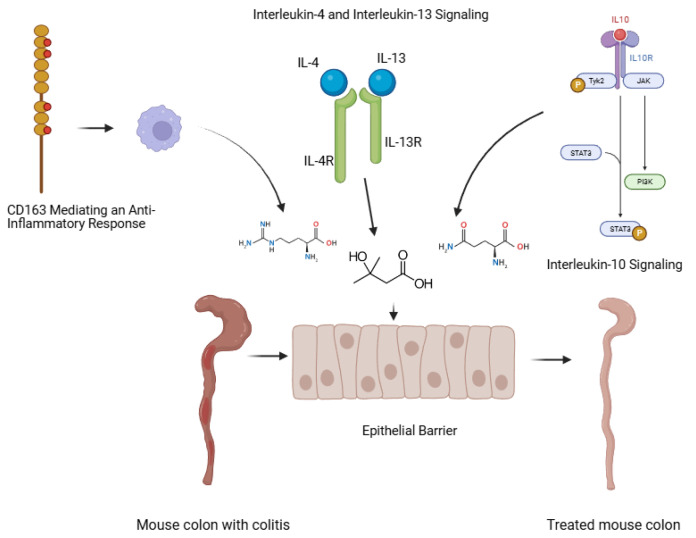
A schematic summary of pathway-level associations identified through gene enrichment analyses. The depicted links between arginine, glutamine, HMB, and immune components reflect functional regulatory relationships inferred from cytokine and metabolic pathway enrichment, rather than direct molecular interactions. These associations are supported by observed gene-expression changes and established immunometabolic mechanisms. Citation to Use: Created in BioRender. TUTAR, Y. (2025) https://BioRender.com/aej03aj.

Enrichment analyses indicated that arginine, glutamine, and HMB-administered individually or in combination—modulate cytokine networks primarily through *IL-10*-related anti-inflammatory signaling, CD163-mediated macrophage responses, and interleukin-4/interleukin-13 pathways. These effects reflect coordinated regulation of immune resolution and tissue repair rather than isolated suppression of pro-inflammatory cytokines (Supplementary Tables S1 and S3, Figure 5).

Like gene expression pattern, the same software provides metabolite interactome (Supplementary Table S2, Supplementary Figure S1). The interaction was visualized in MetaboAnalyst tool. Enrichment of metabolites indicated key pathways involved in treatment (Supplementary Table S4). The results are also in alignment with pathologic experiments.

## 4. Discussion

In this study, we evaluated the effects of arginine, glutamine, and HMB—administered individually or in combination—on inflammatory and metabolic gene expression in DSS-induced colitis. Each amino acid contributed to colitis recovery through distinct but complementary biological pathways: glutamine primarily suppressed inflammatory signaling and enhanced antioxidant defense; arginine modulated nitric oxide–dependent immune regulation; and HMB supported metabolic and mitochondrial adaptation. Combined supplementation integrated these mechanisms, promoting coordinated immunometabolic regulation and mucosal repair. Histopathological examination confirmed typical DSS-induced alterations, including goblet cell loss, glandular disruption, inflammatory cell infiltration, and submucosal edema [21]. Consistent with these findings, the colitis group showed severe mucosal loss and inflammatory granulation tissue, whereas treatment—especially the combination—markedly improved tissue architecture and produced results approaching those of the control group.

Oxidative stress markers responded differentially to treatment. DSS increased MDA and MPO levels and reduced GSH, in agreement with previous reports. Although MPO is primarily an intracellular enzyme stored in neutrophil granules, inflammatory activation and epithelial barrier disruption during DSS-induced colitis promote its extracellular release and systemic translocation. Serum MPO therefore reflects neutrophil-driven oxidative inflammation and increased intestinal permeability, providing complementary information to tissue-level inflammatory assessments. Glutamine significantly elevated GSH and reduced MDA, consistent with its known effects on NF-κB suppression, apoptosis inhibition, and epithelial metabolic support [22,23,24,25]. Arginine also modulated oxidative markers, although outcomes vary across models [9,26,27]. HMB improved antioxidant defense and reduced mucosal inflammation, in line with earlier studies demonstrating its impact on autophagy, apoptosis, and macrophage polarization [13,17,28]. The combination therapy decreased MDA and MPO while partially restoring GSH, suggesting complementary antioxidant actions.

While *IL-6* is a key driver of acute inflammation and was therefore discussed prominently, enrichment and gene-expression data indicate that immunonutrient supplementation also activates *IL-10*–dependent regulatory pathways. Glutamine and HMB promoted *IL-10* expression and anti-inflammatory macrophage responses, whereas arginine influenced cytokine balance through nitric oxide–dependent immune modulation. The combined intervention integrated these mechanisms, resulting in broader cytokine network regulation rather than selective *IL-6* suppression. *IL-6* is a central cytokine in IBD pathogenesis and correlates with disease activity [29,30,31,32]. DSS markedly increased *IL-6* expression, confirming model severity. Glutamine and arginine significantly reduced *IL-6*, consistent with their ability to modulate epithelial repair and cytokine responses [33]. HMB did not suppress *IL-6*, supporting the idea that it influences immune remodeling rather than initial inflammation [13]. In the combination group, *IL-6* remained moderately elevated, suggesting a regulatory rather than suppressive effect when nutrients are co-administered.

*COX2*, induced by *IL-6* and *TNF-α* and elevated in colitis [34], was strongly upregulated in DSS animals. Glutamine and arginine suppressed *COX2*-consistent with their reported inhibition of *COX2*, *iNOS*, and other inflammatory mediators [35,36,37,38]. Although *IL-6* and *COX2* expression remained moderately elevated in the combination group, this finding should not be interpreted as ongoing pathological inflammation. Both mediators exert context-dependent functions and are critically involved in epithelial restitution, angiogenesis, and wound healing during the resolution phase of colitis. The concurrent histopathological improvement reduced oxidative stress markers, and normalization of chemokine signaling suggest that *IL-6* and *COX2* activation in the combination group reflects a regulated reparative response rather than detrimental inflammation. In contrast, HMB and the mixture increased *COX2*, indicating that HMB acts through signaling pathways, such as NF-κB/MAPK, rather than directly inhibiting *COX2* [13,16].

“Given the close interplay between amino acid metabolism, immune regulation, and gut microbial composition, future studies integrating microbiota profiling will be essential to determine whether the observed molecular and histological benefits of these immunonutrients are partially mediated through microbiome-dependent mechanisms.”

*NOS2*/*iNOS* plays a dual role in IBD, contributing to both tissue damage and repair [39,40,41]. Arginine is a substrate for both *NOS2* and *ARG2*, and its effects depend on disease stage [11,34,42]. Increased *ARG2* and *NOS2* in UC patients and DSS mice reflect oxidative stress responses [11,43]. Glutamine reduces *NOS2* expression via NF-κB/MAPK inhibition [33], and HMB may have similar effects [13]. In our study, all treatments increased *NOS2* to varying degrees; HMB induced the strongest expression, suggesting a role in adaptive nitric oxide–mediated repair. The combination group exhibited a more controlled elevation, reflecting regulated NO signaling [44].

Although individual immunonutrients modulated NF-κB and nitric oxide signaling in seemingly divergent ways, these effects are context-dependent and reflect distinct roles in inflammatory initiation, regulation, and resolution. Glutamine primarily attenuated excessive inflammatory and oxidative responses, whereas arginine and HMB contributed to regulated immune signaling, metabolic adaptation, and tissue repair. The combined supplementation integrated these mechanisms, resulting in improved colitis outcomes despite persistence of moderate inflammatory signaling, consistent with a coordinated reparative rather than suppressive response.

*CCR1* regulates immune cell migration to inflamed tissue [45,46,47,48]. DSS markedly increased *CCR1* expression, indicating active leukocyte recruitment. Glutamine strongly reduced *CCR1*, suggesting reduced inflammatory cell mobilization. Arginine and HMB increased *CCR1*, with HMB producing the largest rise. The combination normalized *CCR1* levels, indicating balanced chemokine regulation.

*IL-10* is a key anti-inflammatory cytokine, essential for mucosal tolerance [49,50]. Defective *IL-10* signaling increases *iNOS* activation [39]. Glutamine and HMB increased *IL-10* expression, supporting anti-inflammatory processes [13,34,51]. Arginine showed minimal effect. The combination increased *IL-10*, suggesting enhanced activation of *IL-10*–dependent healing.

*ALDH4A1* participates in amino acid and redox metabolism [52] and may contribute to mucosal integrity [53]. DSS reduced *ALDH4A1*, indicating metabolic stress. All treatments increased *ALDH4A1*, particularly HMB, suggesting improved mitochondrial and redox adaptation. *ALDH4A1*, a mitochondrial enzyme involved in proline degradation and glutamate generation, was markedly upregulated following HMB and combined supplementation. Given its role in redox regulation, mitochondrial metabolism, and aldehyde detoxification, increased *ALDH4A1* expression likely reflects improved metabolic resilience and epithelial adaptation during colitis recovery. The restoration of *ALDH4A1* may contribute to enhanced antioxidant capacity and energy homeostasis, supporting mucosal repair rather than directly suppressing inflammation.

Integration with cytokine signaling and metabolic pathways through enrichment analysis identified *IL-10* signaling, *IL-4/IL-13* signaling, CD163-dependent anti-inflammatory responses, general interleukin signaling, and cytokine signaling in the immune system as key networks activated in colitis. The suppression of *IL-6* and *COX2* by glutamine and arginine, increased *IL-10* in the combination group, and regulated *CCR1* expression align with these cytokine pathways (Supplementary Table S3).

Further, enriched metabolic pathways—arginine/proline metabolism, glutathione metabolism, glutamate metabolism, and nicotinate/nicotinamide pathways—reflect DSS-induced metabolic disruption. Glutamine’s effect on GSH, arginine’s modulation of *NOS2*/*ARG2,* and HMB-induced *ALDH4A1* elevation correspond with restoration of these pathways (Supplementary Table S4).

Metabolite enrichment analysis was used as a supportive, hypothesis-generating tool to contextualize experimentally observed gene-expression and biochemical changes. Only pathways directly aligned with arginine, glutamine, and HMB metabolism and with oxidative stress regulation were interpreted, while broader metabolic associations were not considered mechanistically informative.

Limitations include absence of microbiota profiling, lack of protein-level validation, use of only female rats, lack of dose–response analysis, and absence of long-term outcomes. An important limitation of this study is that molecular analyses were restricted to mRNA expression. Given that transcriptional changes do not always correlate with protein levels or enzymatic activity, future studies incorporating protein quantification, post-translational modifications, and functional assays will be necessary to confirm the biological significance of the observed gene-expression patterns.

Given the pleiotropic and time-dependent nature of NF-κB and nitric oxide signaling, future studies incorporating protein-level analyses, cell-specific resolution, and temporal profiling will be necessary to fully delineate the mechanistic basis of combined immunonutrient action.

Overall, the findings support the therapeutic potential of arginine, glutamine, and HMB as immunonutrients for UC by modulating cytokine networks, restoring metabolic homeostasis, and enhancing epithelial repair.

## 5. Conclusions

This study demonstrates that arginine, glutamine, and HMB each exert distinct yet complementary therapeutic effects in DSS-induced ulcerative colitis. Individually, the amino acids improved selected components of the inflammatory response, oxidative balance, and metabolic stress, whereas the combined supplementation produced a more integrated and balanced modulation across these pathways. Glutamine most effectively improved antioxidant capacity and suppressed *IL6* and *COX2*; arginine contributed to regulation of NO-related metabolism; and HMB enhanced *ALDH4A1* expression and metabolic adaptation. The combination therapy generated broader but more moderate shifts in inflammatory gene expression and restored cytokine and metabolic network coherence, as supported by enrichment analyses.

Together, these findings indicate that immunonutrition with arginine, glutamine, and HMB has therapeutic potential for mitigating colitis by targeting cytokine signaling, chemokine regulation, amino acid metabolism, and oxidative stress defense. Although mechanistic pathways require further confirmation at the protein and clinical levels, this work provides a molecular basis for advancing targeted nutritional strategies in inflammatory bowel disease.

## Figures and Tables

**Table 1:** Relative mRNA Expression Levels in a DSS-Induced Colitis Model After Different Supplementation Regimens.

Genes	UC	UC + Arg	UC + Gln	UC + HMB	UC + Combine
*IL6*	↑12.13 **	↑1.56	↓0.26 **	↑3.51 **	↑1.56
*ALDH4A1*	↓0.69	↑1.44	↑2.13 *	↑6.68 **	↑2.69 **
*ARG2*	↑4.35 **	↓0.60	↓0.50 *	↑2.48 **	↓0.40 *
*CCR1*	↑3.58 **	↑1.40	↓0.36 **	↑4.14 **	↓0.41 **
*COX2*	↑2.68 **	↓0.46 *	↓0.19 **	↑1.65	↑1.76
*NOS2*	↑8.28 **	↑2.91 **	↑2.19 *	↑7.62 **	↑3.38 **
*IL10*	↑1.09	↑1.09	↑1.44	1.00	↑2.15 *

* *p* < 0.05, ** *p* < 0.01. ↑: upregulation, and ↓: downregulation.

## Data Availability

The original contributions presented in this study are included in the article.

## Supplementary Materials

The following supporting information can be downloaded at: https://www.mdpi.com/article/10.3390/nu18020208/s1, Table S1: EnrichR—Reactome Workbench, Table S2: Metabolomics Workbench (EnrichR, MetaboAnalyst 6.0), Table S3: Relationship Between Colitis (IBD) and Key Cytokine/Anti-inflammatory Signaling Pathways, Table S4: Relationship Between Key Metabolic Pathways and Colitis (IBD Pathophysiology), Figure S1: Therapeutic Effect of Arginine, Glutamine and β-Hydroxy β-Methyl Butyrate mixture on Colitis Revealed by Metabolite Enrichment Analysis.

## Abbreviations

The following abbreviations are used in this manuscript:

IBD	Inflammatory bowel diseases
HMB	β-Hydroxy β-methylbutyrate
RT-qPCR	Quantitative Real-Time PCR
MDA	Malondialdehyde
GSH	Glutathione
MPO	Myeloperoxidase
UC	Ulcerative Colitis
