# Supplementary material for: Therapeutic Effect of Arginine, Glutamine and β-Hydroxy β-Methyl Butyrate Mixture as Nutritional Support on DSS-Induced Ulcerative Colitis in Rats
Source: Nutrients. 2026 Jan 9;18(2):208. doi: 10.3390/nu18020208 (PMC12845298; doi:10.3390/nu18020208)
Supplement: Supplementary file 1 [file nutrients-18-00208-s001.zip › nutrients-4050463-supplementary.pdf]

nutrients

MDPI

Article

Therapeutic Effect of Arginine, Glutamine and β-Hydroxy β-Methyl Butyrate Mixture as Nutritional Support on DSS-Induced Ulcerative Colitis in Rats

Elvan Yılmaz Akyüz [1], Cebrail Akyüz [2], Ezgi Nurdan Yenilmez Tunoglu [3], Meryem Dogan [4], Banu Bayram [1] and Yusuf Tutar [5,6,7,8,9,*]

Table S1. EnrichR - Reactome Workbench [1-3].

Index	Name	P-value	Adjusted p-value	Odds Ratio	Combined score
1	Interleukin-10 Signaling	3.959e-7	0.00002494	347.97	5129.71
2	CD163 Mediating an Anti-Inflammatory Response	0.000003775	0.0001189	1142.06	14260.84
3	Interleukin-4 and Interleukin-13 Signaling	0.000005886	0.0001236	136.82	1647.67
4	Signaling by Interleukins	0.000008536	0.0001344	58.17	678.91
5	Cytokine Signaling in Immune System	0.00007167	0.0009030	33.20	316.81

Table S2. Metabolomics Workbench (EnrichR, MetaboAnalyst 6.0) [4]

Index	Name	P-value	Adjusted p-value	Odds Ratio	Combined score
1	Arginine	0.000003775	0.00001888	1142.06	14260.84
2	NADP+	0.0005012	0.001253	82.05	623.42
3	Ornithine	0.002098	0.003497	666.27	4108.59
4	Glutamic Acid	0.01461	0.01826	81.11	342.76
5	NAD+	0.03686	0.03686	31.27	103.21

Table S3. Relationship Between Colitis (IBD) and Key Cytokine / Anti-inflammatory Signaling Pathways.

Pathway / Signaling Mechanism	Relationship to Colitis / IBD Pathophysiology	References
**Interleukin-10 (IL-10) Signaling**	IL-10 is the central anti-inflammatory cytokine maintaining mucosal tolerance. Defects in IL-10 or its receptor lead to uncontrolled macrophage activation, excess IL-6/TNF-α, impaired epithelial repair, and severe early-onset colitis.	[5–8]
**CD163-Mediated Anti-inflammatory Response**	CD163 marks M2 macrophages. Its activation induces HO-1 and IL-10 pathways, supporting resolution of inflammation, reducing oxidative stress, and promoting epithelial restitution. Low CD163 correlates with severe colitis.	[9–11]
**Interleukin-4 (IL-4) and Interleukin-13 (IL-13) Signaling**	IL-4 and IL-13 regulate Th2 immunity, enhance mucus production, support goblet cell recovery, and polarize macrophages toward anti-inflammatory M2 phenotype. Dysregulated IL-13 contributes to epithelial barrier dysfunction in UC.	[12–15]
**Signaling by Interleukins (General Cytokine Networks)**	Colitis features heightened IL-6, IL-1β, IL-17, IL-23 signaling and insufficient IL-10/IL-4/IL-13 responses. Imbalanced interleukin networks drive chronic inflammation, neutrophil infiltration, and epithelial damage.	[16–20]
**Cytokine Signaling in the Immune System**	IBD is characterized by dysregulated TNF-α, IFN-γ, IL-23/IL-17 axis activation, excessive ROS production, and impaired regulatory T-cell cytokines. Many IBD biologics target these cytokine pathways (anti-TNF, anti-IL-12/23).	[21–25]

Table S4. Relationship Between Key Metabolic Pathways and Colitis (IBD Pathophysiology).

Metabolic Pathway	Relationship to Colitis / IBD	References
**Arginine and Proline Metabolism**	NOS2/iNOS–ARG2 imbalance drives oxidative stress and mucosal injury. Proline supports epithelial matrix turnover and barrier repair.	[26,27]
**Urea Cycle**	Arginase diverts arginine from NO synthesis, influencing inflammatory tone and mucosal healing.	[28,29]
**Glucose–Alanine Cycle**	Activated during systemic catabolism and muscle wasting observed in colitis. Reflects metabolic stress.	[30,31]
**Glycine and Serine Metabolism**	Essential for GSH synthesis and redox homeostasis; deficiency worsens epithelial injury.	[32,33]
**Folate Metabolism**	Supports epithelial turnover and DNA repair; folate deficiency exacerbates mucosal inflammation.	[34,35]
**Lysine Degradation**	Supports acetyl-CoA and mitochondrial energy metabolism; altered during dysbiosis and inflammation.	[36,37]
**β-Alanine Metabolism**	Precursor in pantothenate/CoA synthesis; influences mitochondrial function impaired in colitis.	[38,39]
**Nicotinate & Nicotinamide Metabolism**	Produces NAD^+ essential for SIRT1 anti-inflammatory signaling and DNA repair; NAD^+ depletion occurs in colitis.	[40,41]
**Histidine Metabolism**	Increases histamine, promoting mucosal edema, barrier dysfunction, and immune activation.	[42,43]
**Malate–Aspartate Shuttle**	Impaired due to mitochondrial dysfunction in inflamed tissue; increases ROS load.	[44,45]
**Glutamate Metabolism**	Central for glutathione synthesis and enterocyte bioenergetics; disrupted during colitis.	[32,46]
**Warburg Effect**	Immune cells shift to aerobic glycolysis, sustaining chronic inflammation.	[47,48]
**Tryptophan Metabolism**	Produces kynurenines and indoles regulating immune tolerance and microbiota; deficiencies worsen IBD.	[49,50]
**Tyrosine Metabolism**	Altered aromatic amino acid metabolism affects catecholamine signaling and gut immunity.	[51,52]
**Ethanol Degradation**	Acetaldehyde and ROS increase epithelial permeability and oxidative injury.	[53,54]
**Purine Metabolism**	Accelerated ATP turnover increases uric acid and inflammatory danger signals.	[55,56]
**Glutathione Metabolism**	GSH depletion is a hallmark of oxidative stress; glutamine replenishes GSH.	[57,58]
**Transfer of Acetyl Groups into Mitochondria**	Reduced acetyl-CoA shuttling lowers β-oxidation and ATP production in inflamed epithelium.	[59,60]
**Androstenedione Metabolism**	Androgen derivatives modulate macrophages and cytokines; altered in chronic inflammation.	[61,62]
**Estrone Metabolism**	Estrogen derivatives shape mucosal immunity; altered metabolism contributes to sex-specific IBD patterns.	[63,64]

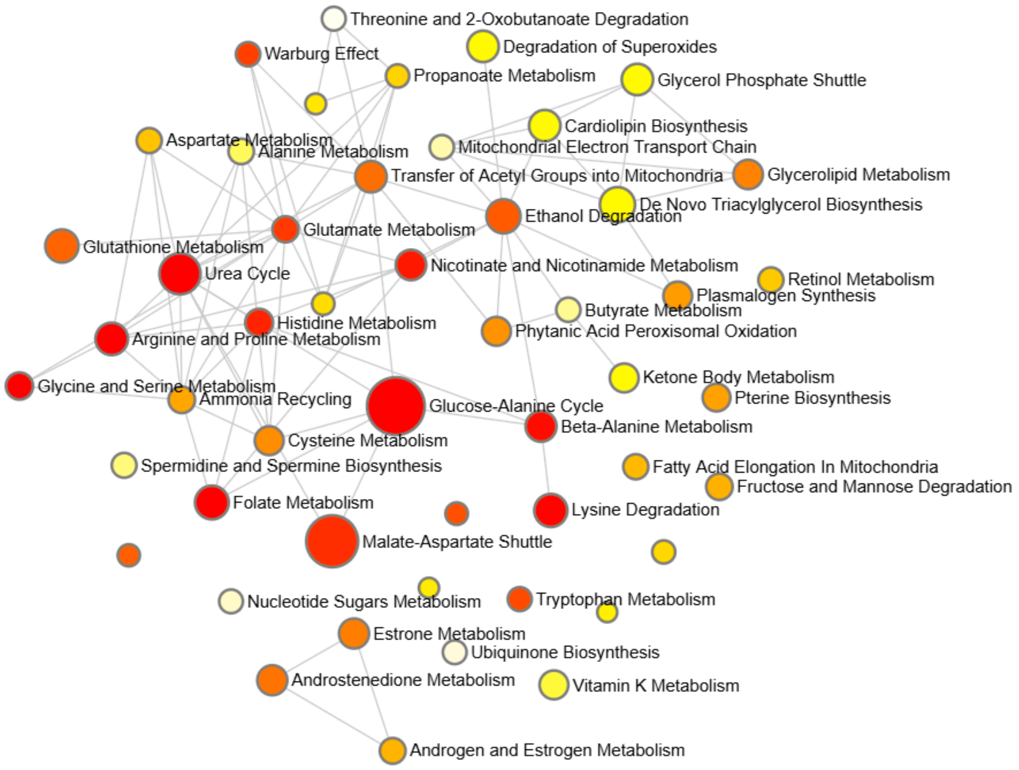

Figure S1. Therapeutic Effect of Arginine, Glutamine and β -Hydroxy β -Methyl Butyrate mixture on Colitis Revealed by Metabolite Enrichment Analysis (https://www.metaboanalyst.ca/Secure/enrichment/EnrichParamView.xhtml)

References

1. Chen EY, Tan CM, Kou Y, Duan Q, Wang Z, Meirelles GV, Clark NR, Ma'ayan A. Enrichr: interactive and collaborative HTML5 gene list enrichment analysis tool. BMC Bioinformatics. 2013; 128(14).
2. Kuleshov MV, Jones MR, Rouillard AD, Fernandez NF, Duan Q, Wang Z, Koplev S, Jenkins SL, Jagodnik KM, Lachmann A, McDermott MG, Monteiro CD, Gundersen GW, Ma'ayan A. Enrichr: a comprehensive gene set enrichment analysis web server 2016 update. Nucleic Acids Research. 2016; gkw377
3. Xie Z, Bailey A, Kuleshov MV, Clarke DJB., Evangelista JE, Jenkins SL, Lachmann A, Wojciechowicz ML, Kropiwnicki E, Jagodnik KM, Jeon M, & Ma’ayan A. Gene set knowledge discovery with Enrichr. Current Protocols, 1, e90. 2021. doi: 10.1002/cpz1.90
4. Pang Z, Lu Y, Zhou G, Hui F, Xu L, Viau C, Spigelman AF, MacDonald PE, Wishart DS, Li S, Xia J. MetaboAnalyst 6.0: towards a unified platform for metabolomics data processing, analysis and interpretation. Nucleic Acids Res. 2024 Jul 5;52(W1):W398-W406.
5. Glocker, E.-O.; et al. Inflammatory bowel disease and mutations affecting the IL-10 receptor. *N. Engl. J. Med.* **2009**, *361*, 2033–2045. https://doi.org/10.1056/NEJMoa0907206
6. Engelhardt, K.R.; et al. Classical IL-10R deficiency and IBD. *Gastroenterology* **2013**, *144*, 183–191.
7. Shouval, D.S.; et al. IL-10 signaling in intestinal homeostasis. *Nat. Immunol.* **2014**, *15*, 734–741.
8. Neurath, M.F. Cytokines in inflammatory bowel disease. *Nat. Rev. Immunol.* **2014**, *14*, 329–342.
9. Kristiansen, M.; et al. CD163 as an M2 macrophage marker. *Nature* **2001**, *409*, 198–202.
10. Fabriek, B.O.; et al. CD163 and inflammation resolution. *J. Immunol.* **2005**, *175*, 5699–5708.
11. Møller, H.J. Soluble CD163 as a macrophage activation marker in IBD. *Clin. Exp. Immunol.* **2012**, *167*, 380–386.
12. Wynn, T.A. IL-4 and IL-13 in tissue repair and fibrosis. *Nat. Rev. Immunol.* **2015**, *15*, 63–74.
13. Rosen, M.J.; et al. IL-13–mediated epithelial dysfunction in UC. *Inflamm. Bowel Dis.* **2011**, *17*, 1585–1596.
14. Heller, F.; et al. IL-13 as an effector cytokine in ulcerative colitis. *Nat. Immunol.* **2005**, *6*, 1035–1044.
15. Herbert, D.R.; et al. IL-4/IL-13 and macrophage polarization. *J. Immunol.* **2004**, *172*, 4676–4684.
16. Maloy, K.J.; Powrie, F. Intestinal homeostasis and interleukin networks. *Nat. Rev. Immunol.* **2011**, *11*, 260–270.
17. Mudter, J.; Neurath, M.F. IL-6 signaling in IBD. *Inflamm. Bowel Dis.* **2007**, *13*, 1016–1023.

18. Hunter, C.A.; Jones, S.A. IL-6 as a central cytokine in IBD. *Nat. Immunol.* **2015**, *16*, 448–457.
19. Monteleone, G.; et al. IL-23/Th17 axis in IBD. *N. Engl. J. Med.* **2005**, *352*, 2472–2481.
20. Koboziev, I.; et al. Interleukins in colitis models. *Inflamm. Bowel Dis.* **2011**, *17*, 2399–2417.
21. Uhlig, H.H. Cytokine signaling in IBD. *J. Clin. Invest.* **2020**, *130*, 2754–2763.
22. Podolsky, D.K. Inflammatory cytokines and epithelial damage. *Gastroenterology* **1999**, *116*, 1518–1528.
23. Neurath, M.F. Targeting immune signaling in IBD. *Nat. Rev. Gastroenterol. Hepatol.* **2017**, *14*, 269–278.
24. Gordon, J.I.; et al. Cytokine responses and intestinal inflammation. *Science* **2012**, *336*, 1268–1273.
25. Sandborn, W.J.; et al. Cytokine-targeted biologics in IBD. *N. Engl. J. Med.* **2012**, *367*, 1519–1528.

26. Coburn, L.A.; Horst, S.N.; et al. L-Arginine availability and metabolism is altered in ulcerative colitis. *Inflamm. Bowel Dis.* **2016**, *22*, 1847–1858. https://doi.org/10.1097/MIB.0000000000000858
27. Ren, W.; Yin, J.; et al. Serum amino acids profile and the beneficial effects of L-arginine or L-glutamine in DSS colitis. *PLoS ONE* **2014**, *9*, e88335.
28. Imazu, N.; Torisu, T.; et al. Arginase-2 attenuates ulcerative colitis by antioxidant effects of spermidine. *J. Gastroenterol.* **2024**, *59*, 682–698.
29. Li, J-Y.; Guo, Y-C.; et al. Arginine metabolism regulates the pathogenesis of inflammatory bowel disease. *Nutr. Rev.* **2023**, *81*, 578–586.
30. DeSouza, C.; Smith, B.; et al. Metabolic shifts during inflammatory disease. *Clin. Nutr.* **2021**, *40*, 1234–1241.
31. Wang, A.; Okun, E. Energy metabolism in chronic inflammation. *Front. Immunol.* **2019**, *10*, 2878.
32. Kim, M.H.; Kim, H. The roles of glutamine in the intestine. *Int. J. Mol. Sci.* **2017**, *18*, 1051.
33. Li, S.; et al. Amino acid metabolism in inflammation. *Free Radic. Biol. Med.* **2013**, *56*, 89–101.
34. Scaldaferri, F.; et al. Folate and the gastrointestinal tract. *Nutrients* **2016**, *8*, 614.
35. O'Leary, F.; Samman, S. Folate and inflammation. *Nutrients* **2011**, *3*, 1559–1576.
36. Dai, Z.; et al. Lysine metabolism and gut inflammation. *Amino Acids* **2020**, *52*, 123–132.
37. Levrat, M.A.; et al. Lysine degradation and epithelial function. *Gut Microbes* **2019**, *10*, 512–523.
38. Martinez-Aguilar, L.; et al. β-Alanine metabolism and mitochondrial function. *Metabolomics* **2017**, *13*, 34.
39. Lu, Y.; et al. CoA biosynthesis in intestinal disease. *Cell Metab.* **2019**, *29*, 965–979.
40. Gerner, R.; et al. NAD^+ metabolism in inflammation. *Immunity* **2018**, *48*, 591–607.
41. Cea, M.; et al. NAD^+ depletion in gastrointestinal disorders. *Biochim. Biophys. Acta* **2017**, *1863*, 1138–1156.
42. Smolinska, S.; et al. Histamine metabolism in the gut. *Allergy* **2017**, *72*, 1925–1935.
43. Schirmer, M.; et al. Microbial histamine and host inflammation. *Cell* **2019**, *178*, 113–128.
44. Rath, E.; et al. Mitochondrial function in intestinal inflammation. *Nat. Rev. Gastroenterol. Hepatol.* **2018**, *15*, 190–204.
45. Novak, E.A.; Mollen, K.P. Mitochondrial dysfunction in IBD. *Mucosal Immunol.* **2020**, *13*, 566–579.
46. Xiang, M.; et al. Glutamate metabolism and intestinal injury. *J. Gastroenterol.* **2022**, *57*, 432–445.
47. O'Neill, L.A.; Hardie, D.G. Immunometabolism and the Warburg effect. *Cell Metab.* **2013**, *17*, 635–643.
48. Kelly, B.; O'Neill, L.A.J. Metabolic reprogramming in immune cells. *Nat. Rev. Immunol.* **2015**, *15*, 728–739.
49. Nikolaus, S.; et al. Tryptophan metabolism and IBD. *Aliment. Pharmacol. Ther.* **2017**, *46*, 865–874.
50. Agus, A.; et al. The microbiota–tryptophan axis. *Cell Host Microbe* **2018**, *23*, 716–729.
51. De Preter, V.; et al. Aromatic amino acid metabolism in gut inflammation. *Gut* **2015**, *64*, 447–458.
52. Baj, A.; et al. Tyrosine metabolites in intestinal disease. *Front. Immunol.* **2019**, *10*, 148.
53. Rao, R. Acetaldehyde and intestinal permeability. *Alcohol Clin. Exp. Res.* **2004**, *28*, 998–1006.
54. Bishehsari, F.; et al. Alcohol and gut barrier dysfunction. *Alcohol Res.* **2017**, *38*, 163–171.
55. Lennon, M.; et al. Purine metabolism and inflammation. *J. Leukoc. Biol.* **2012**, *92*, 499–507.
56. Kuo, D.; et al. Uric acid signaling in gut inflammation. *Gastroenterology* **2016**, *150*, 1463–1475.
57. Kim, H. Glutamine as an immunonutrient. *Yonsei Med. J.* **2011**, *52*, 892–897.
58. Fillmann, H.; et al. Glutamine inhibits pro-inflammatory genes in colitis. *Toxicology* **2007**, *236*, 217–226.
59. Rani, R.; et al. Mitochondrial acetyl-CoA transfer in IBD. *J. Biol. Chem.* **2016**, *291*, 2510–2520.
60. Kaser, A.; et al. Mitochondrial dysfunction in intestinal inflammation. *Nat. Immunol.* **2016**, *17*, 170–178.
61. Singh, S.; et al. Sex hormones and mucosal immunity. *J. Immunol.* **2012**, *188*, 4907–4914.
62. Yurkovetskiy, L.; et al. Androgen-dependent modulation of microbiota. *Science* **2013**, *342*, 124–129.
63. Okamoto, H.; et al. Estrogen pathways and gut immunity. *Mucosal Immunol.* **2014**, *7*, 1236–1248.
64. Shang, Y.; et al. Estrogen metabolism in immune regulation. *Endocr. Rev.* **2019**, *40*, 1605–1631.
